# Assessment of *PAX1* and *JAM3* methylation triage efficacy across HPV genotypes and age groups in high-risk HPV-positive women in China

**DOI:** 10.3389/fonc.2024.1481626

**Published:** 2024-11-26

**Authors:** Hui Liang, Yao Liu, Suyue Yin, Mengyu Jiang, Qiuyan Dou, Hanhan Wang, Jie Liu, Yibo Chen, Pei Liu, Jing Wang, Yishan Wang, Zhe Wu

**Affiliations:** ^1^ Department of Cervical Disease, Xuzhou Maternity and Child Health Hospital, Jiangsu, China; ^2^ Department of Gynecology, Yueyang Central Hospital, Hunan, China; ^3^ Department of Medical Laboratory, Beijing Origin-Poly Bio-Tec Co., Ltd., Beijing, China; ^4^ Department of Clinical Laboratory, Xuzhou Maternity and Child Health Hospital, Jiangsu, China; ^5^ Department of Gynecology, Changsha Women and Child Health Care Hospital affiliated to Hunan Normal University, Hunan, China

**Keywords:** junctional adhesion molecule 3, paired box gene 1, high-risk HPV positive, cervical cancer, *PAX1/JAM3* methylation

## Abstract

**Objective:**

This study aimed to evaluate the clinical utility of *PAX1/JAM3* methylation (CISCER) test in triaging high-risk human papillomavirus (hrHPV)-positive women.

**Methods:**

We enrolled women who underwent opportunistic screening at Cervical Disease outpatient clinics of Xuzhou Maternity and Child Health Hospital, and Yueyang Central Hospital from December 2022 to May 2024. The effectiveness of CISCER and cytology tests in triaging hrHPV+ patients was analyzed.

**Results:**

Among the 436 study participants, 283 (64.9%) had no cervical intraepithelial neoplasia (CIN), while 53 (12.2%) had CIN1, 40 (9.2%) had CIN2, 34 (7.8%) had CIN3, and 26 (5.9%) had cervical cancers. The CISCER tests identified all cases of cervical cancer, particularly 2 hrHPV-negative adenocarcinoma cases. In 396 hrHPV+ individuals, the sensitivity of CISCER tests for detecting CIN2+ lesions was 92.6% (95% CI: 87.2-97.9%), with a specificity of 95.7% (95% CI: 93.4-98%), and an area under the receiver operating characteristic curve (AUC) of 0.941 (95% CI: 0.903-0.979), outperforming cytology tests in both HPV16/18+ and non-16/18 hrHPV+ women. Notably, CISCER demonstrated 100% (95% CI: 90-100%) sensitivity in women aged≥50 and 100% (95%CI: 93.6-100%) specificity in women aged<30. Among CIN2+ women, 37.2% (including 3 cancer) showed low-grade cytological changes that could be detected by CISCER. Meanwhile, 52% of CIN2- women exhibited cytological abnormalities but had negative CISCER results. The immediate CIN3+ risk based on positive CISCER results was 54% (95% CI: 43.8-63.9%).

**Conclusion:**

The *PAX1*/*JAM3* methylation detection using cervical exfoliated cells showed superior triage performance for hrHPV-positive patients compared to traditional strategies.

## Introduction

Globally, cancers of the female reproductive organs (vulva, vagina, cervix, uterus, ovaries) represent approximately 15% of all female cancer cases and deaths (1). In February 2024, the International Agency for Research on Cancer (IARC) reported that 2022 witnessed roughly 1,473,427 new cases and 680,372 deaths in female reproductive organs cancers worldwide (2). Data from China’s National Central Cancer Registry and the World Cancer Research Fund International (WCRF International) for 2022 revealed a persistent increase in new cervical cancer cases in China, with an estimated 150,700 new cases, positioning China as the country with the highest incidence of new cervical cancer cases globally. During the same period, there were approximately 55,700 cervical cancer-related deaths in China (3, 4).

Over 90% of cervical cancers are caused by human papillomavirus (HPV) infection (5). Currently, high-risk HPV (hrHPV) DNA testing is recommended as the primary screening method for cervical cancer in China, with liquid-based cytology testing (LBC) used in resource-limited settings (6). Following infection with hrHPV, patients undergo epigenetic changes that can lead to the development of cervical intraepithelial neoplasia (CIN) 1-3 and, ultimately, cervical cancer (7). HrHPV infection induces mild to moderate cellular abnormalities, which are histologically classified as CIN 1 or CIN 2, whereas most HPV-positive cases are transient and resolve spontaneously, with only a minority progressing to CIN2 or worse (CIN2+). High-grade lesions CIN 2 and CIN 3, both of which are classified by the American Society for Colposcopy and Cervical Pathology (ASCCP) as primary screening targets in precancers, may take decades to progress to cervical cancer (7–9). Thus, HPV positivity for some individuals, especially for women in their 20s, may only signify an infection stage rather than a precancerous state, while misinterpretation of HPV positivity potentially causes undue anxiety, unnecessary treatment, and even increased risk of obstetric complications among screened women (6, 10). Despite high specificity and immediate risk assessment capabilities, cytology’s lower sensitivity and negative predictive value (NPV) limit its effectiveness in long-term risk prediction compared to hrHPV DNA testing (11). While introduction of HPV vaccination has notably reduced cervical cancer caused by vaccine-covered hrHPV genotypes (12–14), the rising incidence of non-vaccine hrHPV genotypes and HPV-unrelated adenocarcinomas is still noteworthy (15, 16). Therefore, within the framework of current guidelines recommending screening methods and comprehensively promoting HPV vaccination, there remains a continuing need to identify appropriate biomarkers as auxiliary screening indicators.

Epigenetic modification mainly involves alterations in gene expression levels driven by non-sequence-based changes. Dysregulated DNA methylation, one of the primary epigenetic mechanisms, contributes considerably to cancer development, invasion, and metastasis (8, 17). Accumulation of DNA methylation in specific genes, acting as an early indicator of malignant transformation, offers new avenues for early prevention, recurrence monitoring, and prognosis assessment (18, 19). Paired box gene 1 (*PAX1*) methylation emerged as a triage tool for detecting CIN3+ among hrHPV-positive women, demonstrating comparable clinical performance with LBC and superior testing efficacy compared to HPV16/18 (20, 21). Additionally, junctional adhesion molecule 3 (*JAM3*) methylation showed good diagnostic accuracy for CIN2+, serving as an effective triage marker for hrHPV-positive patients and stratification method for patients with atypical squamous cells of undetermined significance (ASC-US) (22–24). The combination of these dual genes has also been validated as a tool for cervical cancer screening and stratification. In a multicenter prospective study, *PAX1*/*JAM3* methylation demonstrated superior clinical efficacy, especially the area under the curve (AUC), for testing CIN3+ compared to cytology, leading to a reduction in unnecessary colposcopy referrals (25). Furthermore, elevated *PAX1*/*JAM3* methylation levels correlated significantly with persistent HPV infections lasting over three years, suggesting their potential utility in identifying continuous HPV infection (26). In this report, we conducted a real-world study to evaluate the triage efficacy of *PAX1*/*JAM3* methylation (CISCER) test among hrHPV-positive patients.

## Methods

### Study population

**Figure 1:**
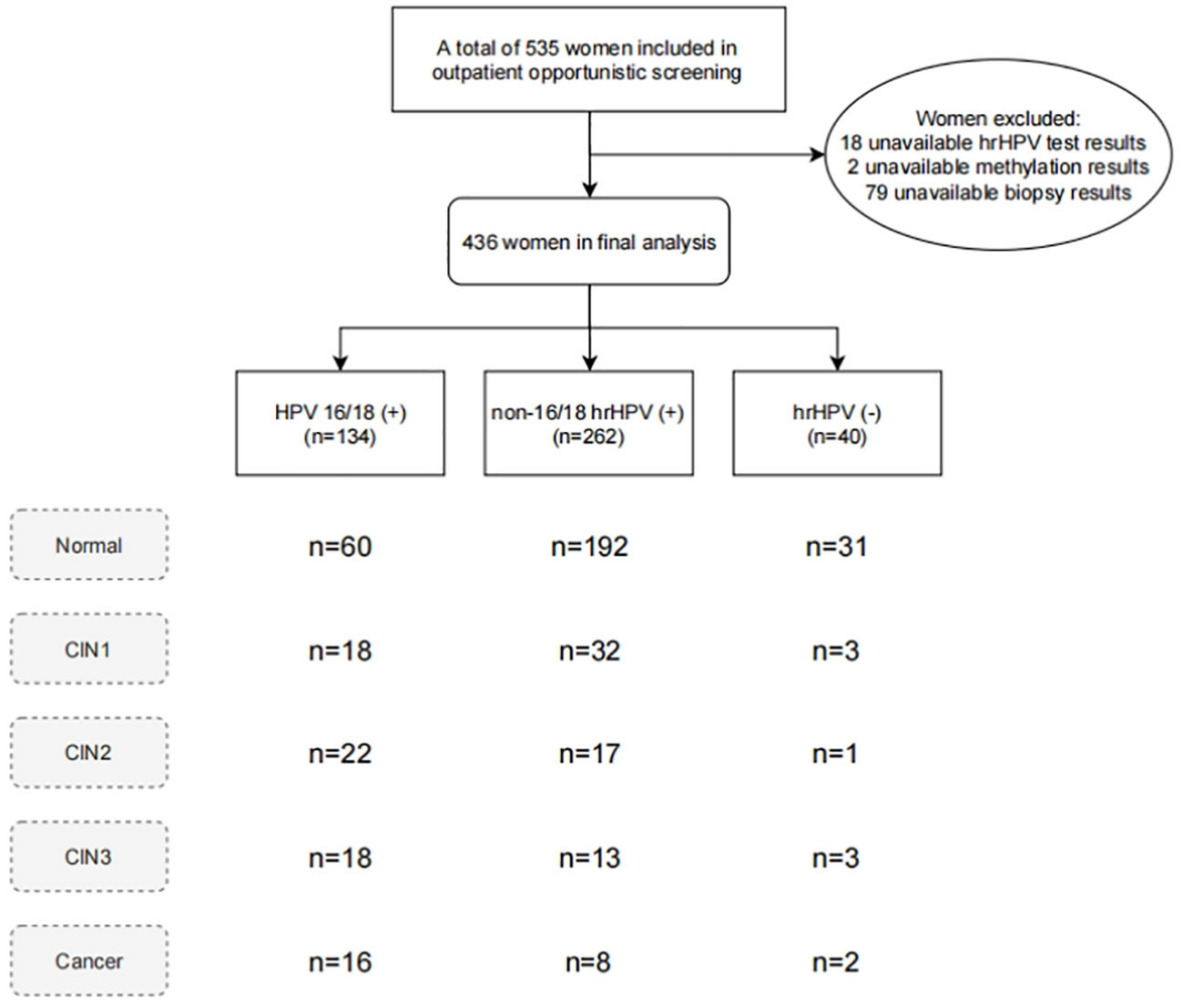
Flowchart of this study.

This study recruited women undergoing opportunistic cervical cancer screening at Cervical Disease outpatient clinics of Xuzhou Maternity and Child Health Hospital, and Yueyang Central Hospital from December 2022 to May 2024. Participants were enrolled based on the following inclusion criteria: 1) age over 18 years; 2) complete medical records and voluntary signing of informed consent. The exclusion criteria were as follows: 1) history of HIV infection or immunodeficiency disorders, and related treatments; 2) sexual activity or vaginal douching in the past 7 days; 3) pregnant or lactating; 4) significant cardiovascular, respiratory, digestive, urinary, hematologic, or psychiatric diseases, and other types of tumors. The study protocol was approved by the ethics committee of Xuzhou Maternity and Child Health Hospital (2023–06) and Yueyang Central Hospital (2024–002). A total of 535 women undergoing cervical screening were included, and the final analysis excluded those without available detection results (**Figure 1**).

### Cytology testing

Cervical exfoliated cell samples were collected using cervical brushes and preserved in cell preservation solution (Landing Intelligent Medicine Co., Ltd, Wuhan, China). These samples were processed into slides using a liquid-based cytology method. Cytology results were classified according to The Bethesda System (TBS) 2014 (27), including categories: negative for intraepithelial lesion or malignancy (NILM), ASC-US, low-grade squamous intraepithelial lesion (LSIL), atypical squamous cells cannot exclude high-grade squamous intraepithelial lesion (ASC-H), high-grade squamous intraepithelial lesion (HSIL), and cervical cancer.

### hrHPV DNA testing

HPV Genotyping Kit for 15 subtypes (Yaneng BIO, Shenzhen, China) was used for genotyping, including 14 hrHPV types: HPV16, HPV18, and 12 other hrHPV types (31, 33, 35, 39, 45, 51, 52, 56, 58, 59, 66 and 68). The collected cervical exfoliated cell samples were stored at 4°C in standard cell media provided by the Kit, and then sent to the laboratory for HPV testing within 48 hours. HPV tests were conducted via polymerase chain reaction (PCR)-reverse dot blot hybridization technology and the testing followed the manufacturer’s instructions as described previously (28).

### DNA isolation and *PAX1*/*JAM3* methylation detection

DNA was isolated from cervical samples using the JH-DNA Isolation and Purifying kit (OriginPoly Bio-Tec Co., Ltd., Beijing, China) according to the manufacturer’s protocol. The DNA concentration was measured with the NanoDrop 2000c spectrophotometer (Thermo Fisher Scientific, DE, USA). Subsequently, 200–1000 ng of DNA underwent bisulfite conversion using the JH-DNA Methylation-Lightning MagPrep kit (OriginPoly Bio-Tec Co., Ltd., Beijing, China).

*PAX1* and *JAM3* gene methylation were assessed using the “Human *PAX1* and *JAM3* Gene Methylation Detection Kit (PCR-fluorescence probe method)” (Origin Biotechnology Co., Ltd., Beijing, China), approved by the National Medical Products Administration as Class III medical device (Registration No. 20233400253). Glyceraldehyde 3-phosphate dehydrogenase (GAPDH) was used as an internal reference (OriGene Technologies, Beijing, China). The analysis was performed using the SLAN-96S fully automated medical PCR analysis system (Hongshi Medical Technology Co., Ltd., Shanghai, China). The PCR reactions started with a single cycle of initial incubation at 96°C for 10 minutes, followed by 45 cycles consisting of denaturation at 94°C for 15 seconds, annealing at 64°C for 5 seconds, and extension at 60°C for 30 seconds. The reaction concluded with a cooling step to 25°C for 1 minute. Clinical personnel were blinded to patient clinical information, LBC results, and cervical tissue pathology results. The hypermethylation status of the *PAX1* and *JAM3* genes was determined by calculating the difference between their respective cycle threshold (Ct) values and that of GAPDH (ΔCt*_PAX1_* = Ct*_PAX1_* - Ct_GAPDH_; ΔCt*_JAM3_* = Ct*_JAM3_* - Ct_GAPDH_). The criteria for defining positive CISCER (*PAX1*/*JAM3* methylation testing) results were ΔCt*_PAX1_* ≤ 6.6 or ΔCt*_JAM3_* ≤10.0, with specific experimental steps and criteria referenced from previous protocols (26). Samples with insufficient DNA concentration for methylation testing were excluded from final analysis.

### Colposcopy and tissue pathology

Patients with abnormal hrHPV DNA or cytology results were referred for colposcopy, and colposcopy-directed biopsies were performed on visible lesions or 1-2 random biopsies were taken from the normal-appearing cervix, unless the patient refused to undergo a biopsy. For women with cervical transformation zone 3 (TZ3), endocervical curettage (ECC) was performed. The positive methylation results were not used as a colposcopy referral indicator.

Histological assessment was performed by two experienced pathologists according to the “Guidelines for cervical cancer screening in China” (6), “Chinese Expert Consensus on Management of Cervical Low-grade Squamous Intraepithelial Lesions” (29), and “Chinese Expert Consensus on Management of Cervical High-grade Squamous Intraepithelial Lesions” (30). Lesion staging included: normal/inflammatory cervical tissue (Normal), CIN1, CIN2, CIN3, squamous cell carcinoma, and adenocarcinoma.

### Statistical analysis

All statistical analyses were conducted using R version 4.4.0 (2024–04–24). The pROC package (version 1.18.4) was used to generate Receiver Operating Characteristic (ROC) curves, AUC, and corresponding 95% Confidence Intervals (95% CI). Sensitivity, specificity, Positive Predictive Value (PPV), and Negative Predictive Value (NPV) with their 95% CI were calculated using functions from the epiR (version 2.0.75) package. Categorical variables were presented as percentages, while continuous variables were presented as medians with interquartile ranges (Q1-Q3). Kruskal-Wallis rank sum test was used for comparing continuous variables between groups, and chi-square tests or Fisher’s exact tests were used for categorical variables. A *P*-value of 0.05 was considered statistically significant.

## Results

### Basic characteristics of patients

A total of 436 women, with a median age of 38 years (range 30 to 49 years), participated in the study (**Table 1**). Histopathological results indicated that among the participants: 298 (66.1%) had normal/inflammatory cervical tissue, followed by 53 (11.8%) with CIN1, 40 (8.9%) with CIN2, 34 (7.5%) with CIN3, 21 (4.7%) with squamous cell carcinoma (SCC), and 5 (1.1%) with adenocarcinoma, totaling 26 cases (5.8%) of cervical cancer. The results of hrHPV DNA testing revealed 40 hrHPV-negative cases, 134 HPV16/18-positive cases, and 262 cases positive for non-16/18 hrHPV. In women who were hrHPV-negative, HPV16/18-positive and non-16/18 hrHPV-positive, the numbers with CIN2+ lesions were 6 (15%), 56 (41.8%), and 38 (14.5%) respectively, with 2 (3.6%), 16 (11.9%), and 8 (3.1%) diagnosed with cervical cancer. This indicates that the proportion of women infected with HPV16/18 developing high grade lesions is significantly higher than those infected with non-16/18 hrHPV (*P* < 0.001), while the proportion of women with abnormal LBC results were lower in women with non-16/18 hrHPV than HPV 16/18. It is noteworthy that two cases of cervical adenocarcinoma had negative hrHPV DNA test results. One of these cases even one showed NILM in LBC results. However, they exhibited high methylation levels of *PAX1* and *JAM3* genes.

**Table 1:** Clinical characteristics.

	Overall (N=436)	hrHPV- (N=40)	HPV16/18(+) (N=134)	non-16/18 hrHPV(+) (N=262)	*P* value
Age					
Median (IQR)	38 (30, 49)	44 (34, 49)	34 (28, 47)	39 (32, 49)	–
Pathology					<0.001
Normal	283 (64.9%)	31 (77.5%)	60 (44.8%)	192 (73.3%)	
CIN1	53 (12.2%)	3 (7.5%)	18 (13.4%)	32 (12.2%)	
CIN2	40 (9.2%)	1 (2.5%)	22 (16.4%)	17 (6.5%)	
CIN3	34 (7.8%)	3 (7.5%)	18 (13.4%)	13 (5.0%)	
Squamous cell carcinoma	21 (4.8%)	0 (0%)	13 (9.7%)	8 (3.1%)	
Adenocarcinoma	5 (1.1%)	2 (5.0%)	3 (2.2%)	0 (0%)	
Cytology					<0.001
NILM	145 (33.3%)	1 (2.5%)	37 (27.6%)	107 (40.8%)	
ASC-US	119 (27.3%)	16 (40.0%)	36 (26.9%)	67 (25.6%)	
LSIL	91 (20.9%)	17 (42.5%)	23 (17.2%)	51 (19.5%)	
ASC-H	35 (8.0%)	3 (7.5%)	9 (6.7%)	23 (8.8%)	
HSIL	40 (9.2%)	2 (5.0%)	26 (19.4%)	12 (4.6%)	
Cervical cancer	6 (1.4%)	1 (2.5%)	3 (2.2%)	2 (0.8%)	
CtPAX1					
Median (IQR)	16.0 (8.6, 17.2)	16.6 (12.5, 17.4)	13.9 (3.8, 17.0)	16.0 (11.3, 17.2)	0.011
CtJAM3					
Median (IQR)	16.0 (13.2, 17.2)	16.5 (13.0, 17.2)	15.2 (5.8, 17.0)	16.0 (14.1, 17.2)	0.036
CISCER					<0.001
Negative	329 (75.5%)	33 (82.5%)	80 (59.7%)	216 (82.4%)	
Positive	107 (24.5%)	7 (17.5%)	54 (40.3%)	46 (17.6%)	

hrHPV-: results of HPV16, 18, 31, 33, 35, 39, 45, 51, 52, 56, 58, 59, 66 and 68 were all negative; HPV16/18(+): positive results for HPV16 and (or) HPV18; non-16/18 hrHPV(+):positive hrHPV results exclusive HPV16/18 positive; CISCER positive criteria: ΔCt*PAX1* ≤ 6.6 and (or) ΔCt*JAM3* ≤ 10.0; CISCER negative criteria: ΔCt*PAX1*>6.6 andΔCt*JAM3*>10.0. N, numbers of subjects; IQR, interquartile range; Normal, normal/inflammatory cervical tissue; CIN, cervical intraepithelial neoplasia; NILM, negative for intraepithelial lesion or malignancy; ASC-US, atypical squamous cells of undetermined significance; LSIL, low-grade squamous intraepithelial lesion; ASC-H, atypical squamous cells cannot exclude high-grade squamous intraepithelial lesion; HSIL, high-grade squamous intraepithelial lesion; HPV, human papillomavirus; hrHPV, high-risk HPV; (+), positive result; (−), negative result.

### The methylation levels of *PAX1* and *JAM3* genes in hrHPV-positive patients

**Figure 2:**
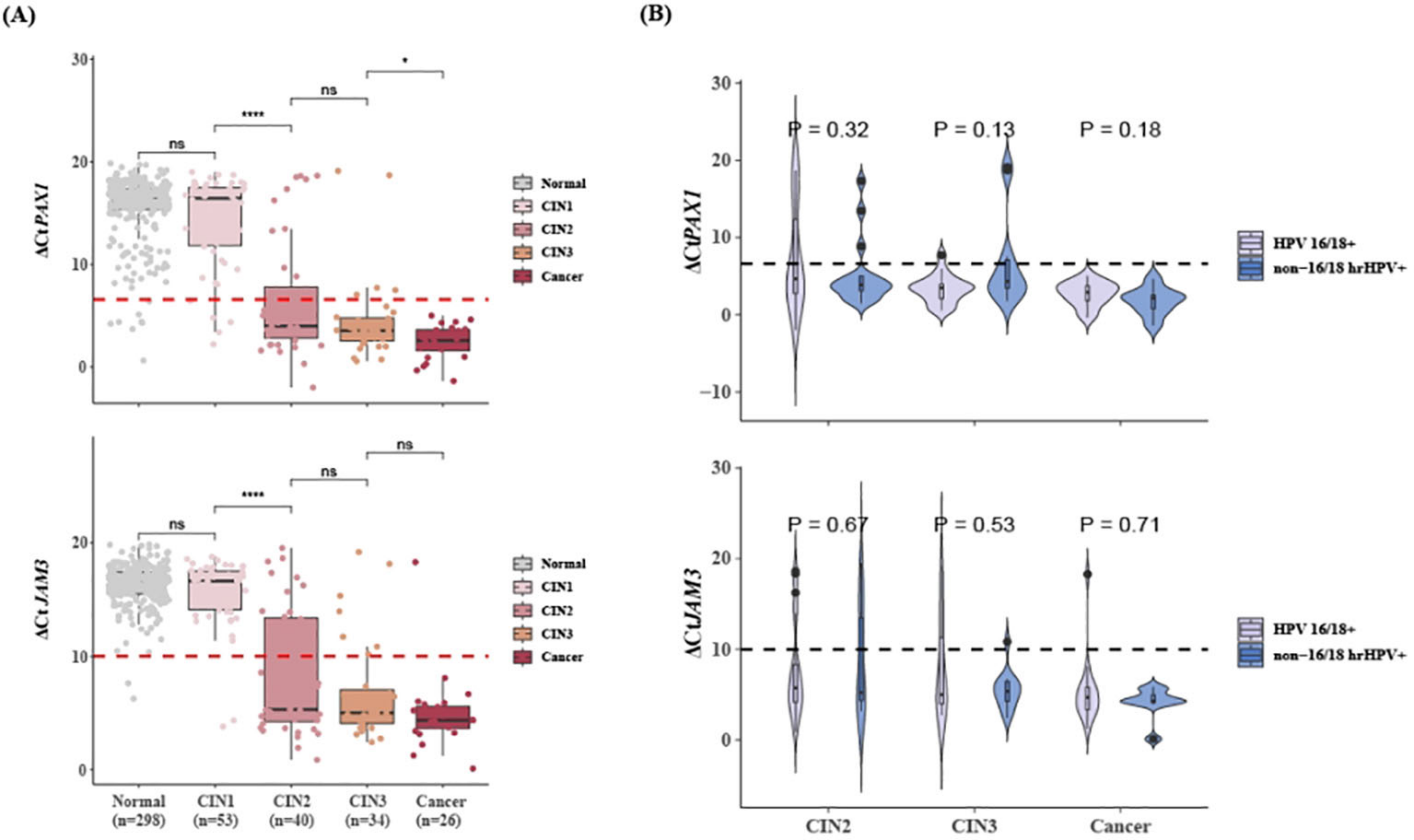
Methylation levels of *PAX1* and *JAM3* gene in hrHPV-positive women with different lesions. **(A)** Distribution plots of ΔCt*PAX1* and ΔCt*JAM3* in hrHPV+ patients with different lesions. **(B)** Distribution plots of ΔCt*PAX1* and ΔCt*JAM3* in CIN2+ patients who infected with HPV 16/18 or non-16/18 hrHPV. Dashed line means ΔCt*PAX1 =* 6.6, ΔCt*JAM3 =* 10.0. `*x*± *s*, ^ns^ *P>*0.05, *P<0.05, ****P<0.0001. *PAX1*, paired box gene 1; *JAM3*, junctional adhesion molecule 3; CIN, cervical intraepithelial neoplasia; hrHPV+, Positive for HPV 16, 18, 31, 33, 35, 39, 45, 51, 52, 56, 58, 59, 66 or 68; non-16/18 hrHPV+, positive hrHPV results excluding HPV16/18.

**Figure 2A** illustrated the ΔCt values of *PAX1* and *JAM3* genes in hrHPV-positive patients with different cervical lesions. The methylation levels of these two genes showed no differences between patients with normal/inflammatory cervical tissue and CIN1, but significantly elevated in CIN2 lesions and continued to increase with disease progression. The mean values of ΔCt*PAX1* and ΔCt*JAM3* in hrHPV-positive patients with CIN2+ were below 6.6 and 10.0, respectively, while they were above these thresholds in those with normal/inflammatory cervical tissue and CIN1. All cervical cancer cases exhibited extremely high methylation levels, and none were missed by CISCER test. Furthermore, we found no statistically significant difference in ΔCt*PAX1* and ΔCt*JAM3* between CIN2+ patients infected with HPV 16/18 and non-16/18 hrHPV (**Figure 2B**). This suggests that once high-grade lesions have developed, the severity of the disease does not differ according to the HPV type.

### Clinical performance of *PAX1*/*JAM3* methylation in hrHPV-positive women

The clinical efficacy of *PAX1* and *JAM3* gene methylation detection in triaging hrHPV-positive women was shown in **Table 2**. Among hrHPV-positive women with CIN2+, CISCER detection demonstrated the highest sensitivity at 92.6% (95% CI: 87.2-97.9%), maintaining a high specificity of 95.7% (95% CI: 93.4-98%). In contrast, LBC_ASC-US+_ had comparable sensitivity (91.5%: 85.8-97.1%) to CISCER tests but markedly lower specificity (45%: 39.4-50.6%), while LBC_ASC-H+_ demonstrated similar specificity (92.7%: 89.8-95.6%) to CISCER tests yet much lower sensitivity (56.4%: 46.4-66.4%). Thus, the CISCER tests achieved a high AUC of 0.941 (95% CI: 0.903-0.979), significantly superior to LBC detection [LBC_ASC-US_+: 0.683(0.626-0.739), LBC_LSIL_: 0.721(0.65-0.791), LBC_ASC-H+_:0.745(0.681-0.81)]. Furthermore, this superiority was observed in women infected with HPV 16/18 and other hrHPV types (**Table 2**, **Supplementary Table S1**).

**Table 2:** Clinical efficacy of different screening methods for triaging hrHPV+ patients.

	Sensitivity% (95%CI)	Specificity% (95%CI)	PPV% (95%CI)	NPV% (95%CI)	AUC (95%CI)	Odds Ratio	P value	Immediate CIN3+ risk (%) for positive results	Immediate CIN3+ risk (%) for negative results
All hrHPV+									
CISCER	92.6 (87.2-97.9)	95.7 (93.4-98)	87 (80.4-93.6)	97.6 (95.9-99.4)	0.941 (0.903-0.979)	276.3 [106.9-714.1]	3.10E-64	54 [43.8-63.9]	0.3 [0-2.2]
PAX1^m^	83 (75.4-90.6)	95.7 (93.4-98)	85.7 (78.5-92.9)	94.8 (92.3-97.3)	0.893 (0.844-0.943)	108.4 [50-234.9]	4.10E-52	54.9 [44.2-65.3]	1.6 [0.6-4]
JAM3^m^	78.7 (70.4-87)	98.7 (97.4-100)	94.9 (90-99.8)	93.7 (91-96.4)	0.887 (0.839-0.935)	275.6 [91.5-830.8]	4.30E-56	60.3 [48.5-71]	2.5 [1.2-5.1]
TCT_ASC-US_	91.5 (85.8-97.1)	45 (39.4-50.6)	34.1 (28.3-40)	94.4 (90.7-98.2)	0.683 (0.626-0.739)	8.8 [4.1-18.8]	8.20E-12	21.4 [16.6-27.1]	0.7 [0-4.4]
TCT_LSIL_	71.3 (62.1-80.4)	72.8 (67.8-77.9)	45 (37-53)	89.1 (85.2-93)	0.721 (0.65-0.791)	6.7 [4-11.1]	3.30E-14	30.9 [23.7-39]	3.6 [1.8-7]
TCT_ASC-H_	56.4 (46.4-66.4)	92.7 (89.8-95.6)	70.7 (60.4-81)	87.2 (83.6-90.9)	0.745 (0.681-0.81)	16.5 [9.1-29.8]	7.90E-23	56 [44.1-67.3]	4 [2.3-7]
HPV 16/18+	59.6 [49.7-69.5]	74.2 [69.2-79.1]	41.8 [33.4-50.1]	85.5 [81.2-89.8]	0.669 [0.594-0.743]	4.2 [2.6-6.9]	4.50E-09	25.4 [18.4-33.8]	8 [5.1-12.2]
Age > =50									
CISCER	100 [80-100]	94.4 [89-99.7]	83.3 [68.4-98.2]	100 [93.2-100]	0.972 [0.945-0.999]	NaN	1.60E-16	62.5 [40.8-80.4]	0 [0-6.8]
TCT_ASC-US_	95 [85.4-100]	50.7 [39.1-62.3]	35.2 [22.4-47.9]	97.3 [92.1-102.5]	0.729 [0.623-0.834]	19.5 [2.5-153.9]	1.80E-04	25.9 [15.4-39.9]	2.7 [0.1-15.8]
HPV 16/18+	65 [44.1-85.9]	81.7 [72.7-90.7]	50 [30.8-69.2]	89.2 [81.7-96.8]	0.733 [0.584-0.883]	8.3 [2.8-24.8]	1.20E-04	34.6 [17.9-55.6]	9.2 [3.8-19.7]
Age < 30									
CISCER	82.6 [67.1-98.1]	100 [93.6-100]	100 [79.1-100]	94.7 [89.6-99.8]	0.913 [0.836-0.99]	NaN	2.50E-16	42.1 [21.1-66]	1.3 [0.1-8.2]
TCT_ASC-US_	82.6 [67.1-98.1]	43.7 [32.1-55.2]	32.2 [20.3-44.1]	88.6 [78-99.1]	0.631 [0.496-0.766]	3.7 [1.1-11.9]	2.70E-02	15.3 [7.6-27.5]	0 [0-12.3]
HPV 16/18+	78.3 [61.4-95.1]	62 [50.7-73.3]	40 [25.7-54.3]	89.8 [81.3-98.3]	0.701 [0.56-0.842]	5.9 [2-17.6]	1.40E-03	15.6 [7-30.1]	4.1 [0.7-15.1]
HPV16/18 +									
CISCER	92.9 (81.9-97.7)	97.4 (90.2-99.6)	96.3 (86.2-99.4)	95.0 (87.0-98.4)	0.951 (0.9-1)	494 [87.3-2796.6]	9.20E-30	61.1 [46.9-73.8]	1.2 [0.1-7.7]
TCT_ASC-US_	91.1 (79.6-96.7)	41.0 (30.2-52.7)	52.6 (42.2-62.7)	86.5 (70.4-94.9)	0.66 (0.569-0.752)	7.1 [2.6-19.7]	3.00E-05	35.1 [25.8-45.5]	0 [0-11.7]
non-16/18 hrHPV+									
CISCER	92.1 (83.5-100)	95.1 (92.3-97.9)	76.1 (63.8-88.4)	98.6 (97.1-100.2)	0.936 (0.879-0.993)	225.9 [60-850.5]	2.50E-30	45.7 [31.2-60.8]	0 [0-2.2]
TCT_ASC-US_	92.1 (83.5-100)	46.4 (39.9-53)	22.6 (16-29.2)	97.2 (94.1-100.3)	0.693 (0.617-0.768)	10.1 [3-33.8]	2.00E-06	12.9 [8.2-19.5]	0.9 [0-5.8]

hrHPV(+): HPV 16, 18, 31, 33, 35, 39, 45, 51, 52, 56, 58, 59, 66 and 68, positive for one or more of them. HPV16/18(+): positive results for HPV16 and (or) HPV18; non-16/18 hrHPV(+):positive hrHPV results exclusive HPV16/18 positive; CISCER positive criteria: ΔCt*PAX1* ≤ 6.6 and (or) ΔCt*JAM3* ≤ 10.0; *PAX1* ^m^ positive criteria: ΔCt*PAX1* ≤ 6.6; *JAM3* ^m^ positive criteria: ΔCt*JAM3* ≤ 10.0; LBC_ASC-US_: the results of cytology were ASC-US and worse, including ASC-US, LSIL, ASC-H, HSIL and cervical cancer; LBC_LSIL_: the results of cytology were LSIL and worse, including LSIL, ASC-H, HSIL and cervical cancer; LBC_ASC-H_: the results of cytology were ASC-H and worse, including ASC-H, HSIL and cervical cancer, CIN2+, cervical intraepithelial neoplasia (CIN) 2 or worse; CI, confidence interval; PPV, positive predictive value; NPV, negative predictive value; AUC, area under the curve; CISCER, *PAX1* ^m^/*JAM3* ^m^; *PAX1* ^m^, the methylation of paired box gene (*PAX1*) gene; *JAM3* ^m^, the methylation of junctional adhesion molecule 3 (*JAM3*) gene; LBC, liquid-based thin-layer cytology testing; (+), positive result; (−), negative result.

The immediate CIN3+ risk based on positive CISCER results would be 54% (95% CI: 43.8-63.9%), significantly higher than that of LBC_ASC-US_ (21.4%: 16.6-27.1%) and LBC_LSIL_ (30.9%: 23.7-39%). When the cytology test result showed ASC-H, HSIL or cancer, the patient’s risk of CIN3+ was 56% (95%CI: 44.1-67.3%), slightly higher than CISCER (54%: 43.8-63.9). Conversely, with LBC results of NILM, ASC-US or LSIL, the risk of CIN3+ was 4% (95%CI: 44.1-67.3%), markedly higher than that of CISCER-negative (0.3%: 0-2.2%). This suggests that low-grade cytology results could experience 4% risk of missing the diagnosis of CIN3+, a risk that CISCER can effectively mitigate.

Notably, the sensitivity of CISCER tests achieved 100% (95% CI: 80-100%) in women aged over 50, with a high specificity of 94.4% (95% CI: 94.4-99.7%). In this demographic, a positive CISCER result indicated a 62.5% risk of CIN3+ (95% CI: 40.8-80.4%), while a negative result reduces the risk of CIN3+ to 0%. Additionally, CISCER achieved 100% specificity in women under 30, minimizing unnecessary interventions and protecting fertility in this younger population (More information in **Supplementary Table S1**).

### Comparison of *PAX1*/*JAM3* methylation and cytology detection

**Figure 3:**
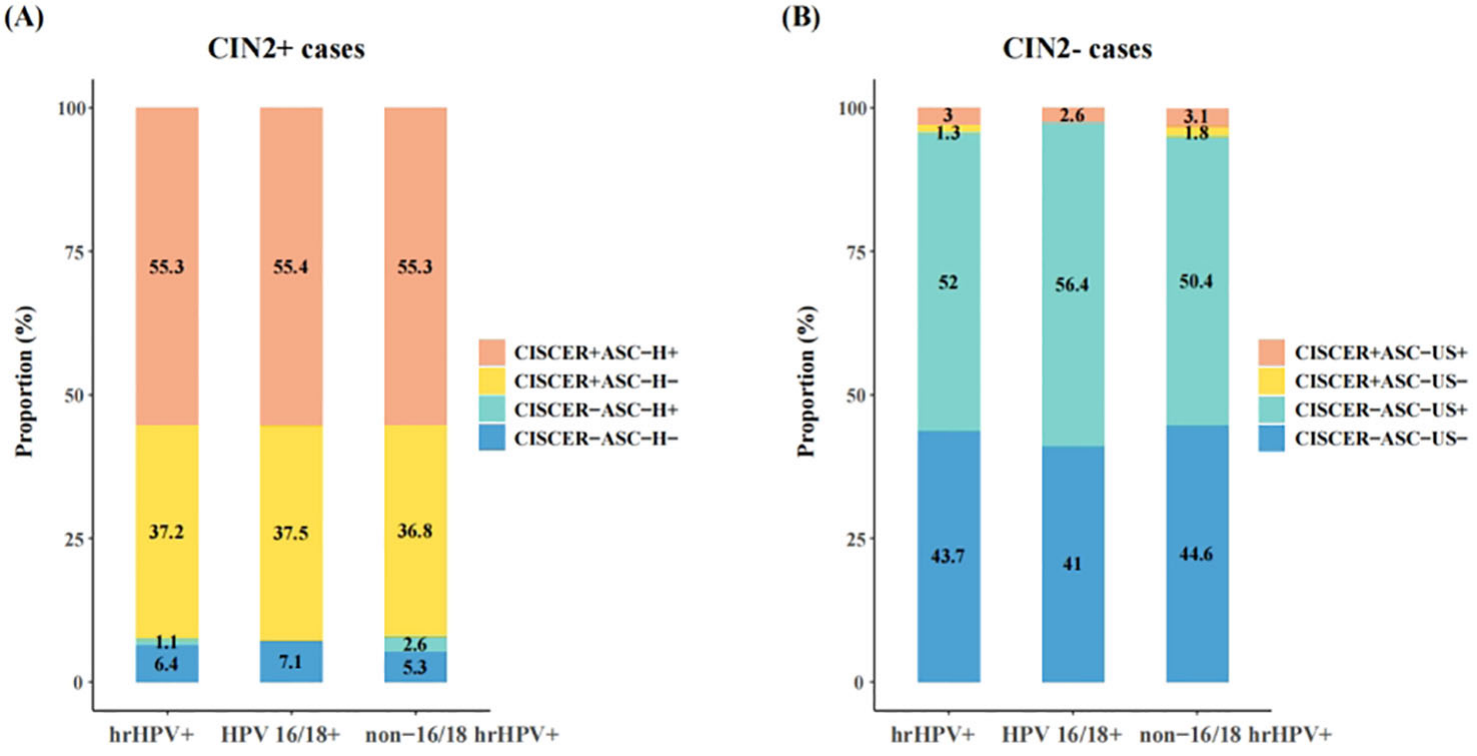
Comparison between CISCER and cytology results in hrHPV+ patients with CIN2+ or CIN2- lesions. **(A)** Results of CISCER and cytology tests in hrHPV+ patients with CIN2+ lesions. **(B)** Results of CISCER and cytology tests in hrHPV+patients with CIN2- lesions. CIN, cervical intraepithelial neoplasia; CIN2+ cases, patients with CIN 2 or severe lesions; CIN2- cases, patients with normal/inflammatory cervical tissue or CIN1; ASC-H-, the results of cytology were NILM, ASC-US or LSIL; ASC-H+, the results of cytology were ASC-H, HSIL or cervical cancer; CISCER+, ΔCt*PAX1* ≤ 6.6 and (or) ΔCt*JAM3* ≤ 10.0; CISCER-, ΔCt*PAX1*>6.6 and ΔCt*JAM3*>10.0; ASC-US-, the results of cytology were NILM; ASC-US+, the results of cytology were ASC-US, LSIL, ASC-H, HSIL or cervical cancer.

We further analyzed the proportions of women based on their CISCER test and LBC results, stratified for histology (**Figure 3**). In hrHPV-positive patients with CIN2+ lesions (including one cervical squamous carcinoma and two adenocarcinoma cases), approximately 45% (43.6%) showed cytology results of NILM, ASC-US or LSIL, whereas the CISCER test yielded positive results in 37.2% of these women, underscoring the potential of methylation tests to capture a substantial proportion of missed diagnoses caused by inaccurate cytological results (**Figure 3A**). In addition, this proportion was 37.5% for women positive for HPV 16/18 and 36.8% for those positive for non-16/18 hrHPV (**Figure 3A**).

On the other hand, abnormal LBC results (ASC-US+) were observed in 55% of women with normal/inflammatory cervical tissue or CIN1, which may lead to unnecessary medical resource utilization and reduced patient compliance if all these women were referred to colposcopy or treatment (**Figure 3B**). The high specificity of CISCER tests effectively mitigated this issue, achieving negative results in 52% of these cases (**Figure 3B**). These findings once again underscore the CISCER test can serve as a powerful tool for stratifying hrHPV-positive women without relying on assessment of cellular morphology.

## Discussion

This study evaluated the clinical efficacy of *PAX1*/*JAM3* methylation detection in triaging HPV16/18-positive and non-16/18 hrHPV-positive patients within a cohort of 436 enrolled women, including 396 who were hrHPV-positive. Our findings demonstrated robust performance of CISCER test with sensitivity and specificity exceeding 92%. In addition, *PAX1* and *JAM3* methylation detection, whether alone or in combination, achieved a more balanced sensitivity and specificity profile and higher AUC values compared to these of cytology. Importantly, our study highlighted that the CISCER tests successfully identified all cervical cancer cases among the enrolled patients, including two adenocarcinoma cases that were hrHPV- negative.

Cervical cancer stands as the most predominant malignancy affecting the female reproductive system and a leading cause of female cancer-related deaths worldwide, accounting for approximately 6.9% of all cancer cases and 8.1% of fatalities as of February 2024 (2). Recent data from the National Cancer Center of China reveal a rising trend in cervical cancer incidence and mortality rate among Chinese women from 2010 to 2018 (4). Adding to the problem, cervical cancer screening coverage remains inadequate, with only 36.8% of women aged 35-64 undergoing screening between 2018 and 2019 in China, particularly in rural areas, as well as in the central and western regions (31). While cytology offers high specificity, its limited sensitivity (50-70%) poses challenges in detecting high-grade lesions and contributes to potential underdiagnosis issues (32, 33). Since 97% of precancerous lesions are HPV-positive, there is limited additional information that can be provided by performing both cytology and HPV DNA testing compared to HPV DNA testing alone (34, 35).

DNA methylation has garnered international recognition as a pivotal tool in cervical cancer screening, triage, and management, due to its reliable performance in efficiently detecting precancerous lesions (7, 36, 37). The latest expert consensus released by the Tumor marker committee of Chinese anti-cancer association in 2024 proposes that DNA methylation testing could play a role in cervical cancer screening management and post-treatment monitoring (38). Previous research, as evidenced by study including conditional inference tree model, had shown that methylation assay was more pertinent to the pathological diagnosis than cytology in diagnosing both high- and low-grade cervical lesions, and the assay can effectively differentiate high-grade cervical lesions regardless of cytology test (39). DNA methylation testing could mitigate overtreatment in patients diagnosed as CIN2+, where a positive result for specific methylation markers indicates a heightened risk of short-term progression to cervical cancer (7). In this study, we observed that over 35% of CIN2+ patients had negative cytology or low-grade lesions (ASC-US and LSIL) but positive CISCER results, including 3 cancer cases. Additionally, more than 50% of CIN2- patients was abnormal cytology (ASC-US+) but negative for CISCER tests. Moreover, CISCER’s immediate CIN3+ risk for negative results in hrHPV-positive women was much lower than LBC. These finding indicates that CISCER tests held a lower missed diagnosis rate in high-grade lesions and also resulted in fewer referrals for colposcopy in women with low-grade lesions. This highlights the potential of *PAX1*/*JAM3* methylation to complement hrHPV tests in triaging HPV-positive women rather than relying on cytology detection.

Cervical cancer is frequently diagnosed in populations lacking adequate screening (40). Despite hrHPV DNA test being recommended as the primary screening tool for cervical cancer (6, 41), not all cases are associated with HPV infection, such as gastric-type endocervical adenocarcinoma (G-EAC). In our study cohort, two adenocarcinoma patients tested negative for hrHPV DNA test, but were successfully identified by CISCER tests. Specifically, these two cases exhibited notably high levels of *PAX1* and *JAM3* methylation (Patient 1: ΔCt*PAX1 =* 2.52, ΔCt*JAM3 =* 5.28, LBC: NILM; Patient 2: ΔCt*PAX1 =* 1.82, ΔCt*JAM3 =* 3.72, LBC: adenocarcinoma). Thus, DNA methylation tests in our study provided more comprehensive identification of cervical cancer patients compared to hrHPV DNA tests, consistent with previous findings (42). Therefore, *PAX1*/*JAM3* methylation detection could not only reduce the number of patients who were missed or misdiagnosed by cytology, but also offers a highly sensitive marker panel for cancer, especially those unrelated to HPV infection.

We observed that CISCER had higher sensitivity in women aged≥50 and higher specificity in women aged<30. An analysis of cervical cancer trends among Chinese women from 1990 to 2019 reveals that the highest incidence and mortality rates were observed in the 50-54 age group, followed by women aged 55 and older (43). This pattern may be influenced by hormonal changes in postmenopausal women, which lead to genital tract atrophy and adhesions and then complicate the collection of adequate samples during gynecological examinations (44–46), which may cause anxiety in healthy women. Even worse, the squamous-columnar junction of the cervix shifts upward for elderly women, causing common sites of cervical cancer and precancerous lesions to retract into the cervical canal. This anatomical change often results in missed or inaccurate diagnoses during cervical cytology, colposcopy biopsy, or endocervical curettage (ECC), leading to delays in the initiation of appropriate treatment (47). These changes have introduced significant challenges for cervical cancer screening in postmenopausal women. The high sensitivity of CISCER tests in our study suggests its effectiveness in accurately identifying high-risk patients, which is essential for optimizing cervical cancer screening strategies for postmenopausal women.

Efforts to eliminate cervical cancer in China include extensive HPV vaccination campaigns nationwide, aiming at increasing vaccination rates among women (48, 49). A national survey identified HPV 52, 58, 53, 16, and 51 as the predominant genotypes, varying significantly by age groups and geographic locations in China (50). HPV vaccination efforts expand, necessitating effective cervical cancer screening methods for vaccinated and non-vaccinated individuals and also those infected with non-vaccinated hrHPV genotypes. A long-term follow-up of Dutch women vaccinated with the bivalent vaccine in adolescence revealed high-grade cervical lesions were still detectable in women who were vaccinated early, and the main types of HPV infection were HPV52, 59, 51, 58, and 33, which were non-vaccinated HPV genotypes (12). We observed consistent performance of *PAX1* and *JAM3* methylation in screening hrHPV-positive patients with CIN2+ regardless of HPV16/18 or non-16/18 hrHPV infections, and *PAX1*/*JAM3* methylation levels were comparable between high-grade cervical lesions caused by non-16/18 hrHPV and HPV16/18. Given the direct correlation where higher levels of methylation correspond to more advanced cervical disease (51), it suggested that the severity of cervical disease did not vary based on hrHPV genotypes once high grade lesions had emerged. Hence, cervical diseases caused by non-16/18 hrHPV genotypes are also noteworthy, as women may not experience reduced severity of cervical lesions due to infection with these genotypes. Methylation tests hold the ability to detect high-grade cervical lesions caused by non-vaccinated hrHPV genotypes, which underscores the potential for them to perform well in screening programs as more vaccinated individuals enter the screening cohort in the future.

Limitations of our study included its simple size, which might limit the generalizability of the findings due to the lack of extensive research data supporting the results. In addition, the histopathological results relied on biopsies from patients referred to colposcopy outpatients rather than surgical pathological results, potentially impacting the accuracy of the histopathological assessments. Future multicenter prospective studies with larger sample sizes and rigorous clinical evaluations are needed to further validate the clinical utility of DNA methylation testing in cervical cancer screening.

## Conclusions

Our findings indicate that *PAX1*/*JAM3* methylation test significantly outperforms cytology in triaging hrHPV-positive patients. This could help mitigate the issue of missed diagnoses in postmenopausal women and address fertility preservation concerns in younger women. Furthermore, *PAX1*/*JAM3* methylation test could be valuable for detecting cervical lesions associated with non-vaccine HPV types.

## Data Availability

The raw data supporting the conclusions of this article will be made available by the authors, without undue reservation.
