# Supplementary material for: Assessment of PAX1 and JAM3 methylation triage efficacy across HPV genotypes and age groups in high-risk HPV-positive women in China
Source: Front Oncol. 2024 Nov 26;14:1481626. doi: 10.3389/fonc.2024.1481626 (PMC11628385; doi:10.3389/fonc.2024.1481626)
Supplement: Supplementary file 1 [file Table1.docx]

**Table S1 Clinical efficacy of different screening methods for triaging hrHPV+ patients**

|  | **Sensitivity%**  **(95%CI)** | **Specificity%**  **(95%CI)** | **PPV%**  **(95%CI)** | **NPV%**  **(95%CI)** | **AUC**  **(95%CI)** | **Odds Ratio** | **P value** | **Immediate CIN3+ risk**  **(%) for positive results** | **Immediate CIN3+ risk**  **(%) for negative results** |
| --- | --- | --- | --- | --- | --- | --- | --- | --- | --- |
| **All hrHPV+** |  |  |  |  |  |  |  |  |  |
| CISCER | 92.6  [87.2-97.9] | 95.7  [93.4-98] | 87  [80.4-93.6] | 97.6  [95.9-99.4] | 0.941  [0.903-0.979] | 276.3 [106.9-714.1] | 3.10E-64 | 54  [43.8-63.9] | 0.3  [0-2.2] |
| PAX1^m^ | 83  [75.4-90.6] | 95.7  [93.4-98] | 85.7  [78.5-92.9] | 94.8  [92.3-97.3] | 0.893  [0.844-0.943] | 108.4 [50-234.9] | 4.10E-52 | 54.9  [44.2-65.3] | 1.6  [0.6-4] |
| JAM3^m^ | 78.7  [70.4-87] | 98.7  [97.4-100] | 94.9  [90-99.8] | 93.7  [91-96.4] | 0.887  [0.839-0.935] | 275.6 [91.5-830.8] | 4.30E-56 | 60.3  [48.5-71] | 2.5  [1.2-5.1] |
| TCT_ASC-US_ | 91.5  [85.8-97.1] | 45  [39.4-50.6] | 34.1  [28.3-40] | 94.4  [90.7-98.2] | 0.683  [0.626-0.739] | 8.8 [4.1-18.8] | 8.20E-12 | 21.4  [16.6-27.1] | 0.7  [0-4.4] |
| TCT_LSIL_ | 71.3  [62.1-80.4] | 72.8  [67.8-77.9] | 45  [37-53] | 89.1  [85.2-93] | 0.721  [0.65-0.791] | 6.7 [4-11.1] | 3.30E-14 | 30.9  [23.7-39] | 3.6  [1.8-7] |
| TCT_ASC-H_ | 56.4  [46.4-66.4] | 92.7  [89.8-95.6] | 70.7  [60.4-81] | 87.2  [83.6-90.9] | 0.745  [0.681-0.81] | 16.5 [9.1-29.8] | 7.90E-23 | 56  [44.1-67.3] | 4  [2.3-7] |
| HPV 16/18+ | 59.6  [49.7-69.5] | 74.2  [69.2-79.1] | 41.8  [33.4-50.1] | 85.5  [81.2-89.8] | 0.669  [0.594-0.743] | 4.2 [2.6-6.9] | 4.50E-09 | 25.4  [18.4-33.8] | 8  [5.1-12.2] |
| **Age > =50** |  |  |  |  |  |  |  |  |  |
| CISCER | 100  [80-100] | 94.4  [89-99.7] | 83.3  [68.4-98.2] | 100  [93.2-100] | 0.972  [0.945-0.999] | NaN | 1.60E-16 | 62.5  [40.8-80.4] | 0  [0-6.8] |
| PAX1^m^ | 100  [80-100] | 94.4  [89-99.7] | 83.3  [68.4-98.2] | 100  [93.2-100] | 0.972  [0.945-0.999] | NaN | 1.60E-16 | 62.5  [40.8-80.4] | 0  [0-6.8] |
| JAM3^m^ | 90  [76.9-100] | 98.6  [95.9-100] | 94.7  [84.7-104.8] | 97.2  [93.4-101] | 0.943  [0.864-1] | 630 [54.1-7342.4] | 7.40E-16 | 73.7  [48.6-89.9] | 1.4  [0.1-8.5] |
| TCT_ASC-US_ | 95  [85.4-100] | 50.7  [39.1-62.3] | 35.2  [22.4-47.9] | 97.3  [92.1-102.5] | 0.729  [0.623-0.834] | 19.5 [2.5-153.9] | 1.80E-04 | 25.9  [15.4-39.9] | 2.7  [0.1-15.8] |
| TCT_LSIL_ | 85  [69.4-100] | 78.9  [69.4-88.4] | 53.1  [35.8-70.4] | 94.9  [89.3-100.5] | 0.819  [0.694-0.945] | 21.2 [5.5-81.9] | 2.90E-07 | 40.6  [24.2-59.2] | 3.4  [0.6-12.7] |
| TCT_ASC-H_ | 75  [56-94] | 91.5  [85.1-98] | 71.4  [52.1-90.8] | 92.9  [86.8-98.9] | 0.833  [0.706-0.96] | 32.5 [8.7-120.8] | 1.00E-08 | 57.1  [34.4-77.4] | 4.3  [1.1-12.8] |
| HPV 16/18+ | 65  [44.1-85.9] | 81.7  [72.7-90.7] | 50  [30.8-69.2] | 89.2  [81.7-96.8] | 0.733  [0.584-0.883] | 8.3 [2.8-24.8] | 1.20E-04 | 34.6  [17.9-55.6] | 9.2  [3.8-19.7] |
| **Age < 30** |  |  |  |  |  |  |  |  |  |
| CISCER | 82.6  [67.1-98.1] | 100  [93.6-100] | 100  [79.1-100] | 94.7  [89.6-99.8] | 0.913  [0.836-0.99] | NaN | 2.50E-16 | 42.1  [21.1-66] | 1.3  [0.1-8.2] |
| PAX1^m^ | 65.2  [45.8-84.7] | 100  [93.6-100] | 100  [74.7-100] | 89.9  [83.2-96.5] | 0.826  [0.729-0.923] | NaN | 5.30E-12 | 46.7  [22.3-72.6] | 2.5  [0.4-9.7] |
| JAM3^m^ | 65.2  [45.8-84.7] | 100  [93.6-100] | 100  [74.7-100] | 89.9  [83.2-96.5] | 0.826  [0.729-0.923] | NaN | 5.30E-12 | 40  [17.5-67.1] | 3.8  [1-11.5] |
| TCT_ASC-US_ | 82.6  [67.1-98.1] | 43.7  [32.1-55.2] | 32.2  [20.3-44.1] | 88.6  [78-99.1] | 0.631  [0.496-0.766] | 3.7 [1.1-11.9] | 2.70E-02 | 15.3  [7.6-27.5] | 0  [0-12.3] |
| TCT_LSIL_ | 52.2  [31.8-72.6] | 73.2  [62.9-83.5] | 38.7  [21.6-55.9] | 82.5  [73.2-91.9] | 0.627  [0.474-0.781] | 3 [1.1-7.9] | 4.00E-02 | 22.6  [10.3-41.5] | 3.2  [0.6-12] |
| TCT_ASC-H_ | 30.4  [11.6-49.2] | 93  [87-98.9] | 58.3  [30.4-86.2] | 80.5  [71.9-89.1] | 0.617  [0.493-0.741] | 5.8 [1.6-20.6] | 7.80E-03 | 33.3  [11.3-64.6] | 6.1  [2.3-14.3] |
| HPV 16/18+ | 78.3  [61.4-95.1] | 62  [50.7-73.3] | 40  [25.7-54.3] | 89.8  [81.3-98.3] | 0.701  [0.56-0.842] | 5.9 [2-17.6] | 1.40E-03 | 15.6  [7-30.1] | 4.1  [0.7-15.1] |
| **HPV16/18 +** |  |  |  |  |  |  |  |  |  |
| CISCER | 92.9  [81.9-97.7] | 97.4  [90.2-99.6] | 96.3  [86.2-99.4] | 95.0  [87.0-98.4] | 0.951  [0.9-1] | 494 [87.3-2796.6] | 9.20E-30 | 61.1  [46.9-73.8] | 1.2  [0.1-7.7] |
| PAX1^m^ | 83.9  [71.2-91.9] | 97.4  [90.2-99.6] | 95.9  [84.9-99.3] | 89.4  [80.4-94.7] | 0.907  [0.841-0.972] | 198.4 [41.1-958.4] | 2.00E-24 | 67.3  [52.3-79.6] | 1.2  [0.1-7.3] |
| JAM3^m^ | 78.6  [65.2-88.0] | 98.7  [92.1-99.9] | 97.8  [86.8-99.9] | 86.5  [77.2-92.5] | 0.886  [0.82-0.953] | 282.3 [35.5-2244.9] | 4.30E-23 | 60  [44.4-73.9] | 7.9  [3.5-16.1] |
| TCT_ASC-US_ | 91.1  [79.6-96.7] | 41.0  [30.2-52.7] | 52.6  [42.2-62.7] | 86.5  [70.4-94.9] | 0.66  [0.569-0.752] | 7.1 [2.6-19.7] | 3.00E-05 | 35.1  [25.8-45.5] | 0  [0-11.7] |
| TCT_LSIL_ | 73.2  [59.5-83.8] | 74.4  [63.0-83.3] | 67.2  [53.9-78.4] | 79.5  [68.1-87.7] | 0.738  [0.631-0.844] | 7.9 [3.6-17.3] | 7.20E-08 | 47.5  [34.8-60.6] | 6.8  [2.5-15.9] |
| TCT_ASC-H_ | 55.4  [41.6-68.4] | 91.0  [81.8-96.0] | 81.6  [65.1-91.7] | 74.0  [63.8-82.1] | 0.732  [0.635-0.829] | 12.6 [4.9-32.1] | 4.50E-09 | 68.4  [51.2-82] | 8.3  [3.9-16.2] |
| **non-16/18 hrHPV+** |  |  |  |  |  |  |  |  |  |
| CISCER | 92.1  [83.5-100] | 95.1  [92.3-97.9] | 76.1  [63.8-88.4] | 98.6  [97.1-100.2] | 0.936  [0.879-0.993] | 225.9 [60-850.5] | 2.50E-30 | 45.7  [31.2-60.8] | 0  [0-2.2] |
| PAX1^m^ | 81.6  [69.3-93.9] | 95.1  [92.3-97.9] | 73.8  [60.5-87.1] | 96.8  [94.5-99.1] | 0.883  [0.808-0.959] | 85.8 [30.9-237.8] | 2.20E-24 | 40.5  [26-56.7] | 1.8  [0.6-4.9] |
| JAM3^m^ | 78.9  [66-91.9] | 98.7  [97.2-100] | 90.9  [81.1-100.7] | 96.5  [94.1-98.9] | 0.888  [0.816-0.96] | 276.2 [69.5-1098.7] | 1.00E-28 | 60.6  [42.2-76.6] | 0.4  [0-2.8] |
| TCT_ASC-US_ | 92.1  [83.5-100] | 46.4  [39.9-53] | 22.6  [16-29.2] | 97.2  [94.1-100.3] | 0.693  [0.617-0.768] | 10.1 [3-33.8] | 2.00E-06 | 12.9  [8.2-19.5] | 0.9  [0-5.8] |
| TCT_LSIL_ | 68.4  [53.6-83.2] | 72.3  [66.5-78.2] | 29.5  [20-39.1] | 93.1  [89.3-96.9] | 0.704  [0.601-0.807] | 5.7 [2.7-11.9] | 3.10E-06 | 19.3  [12-29.4] | 2.3  [0.7-6.2] |
| TCT_ASC-H_ | 57.9  [42.2-73.6] | 93.3  [90-96.6] | 59.5  [43.6-75.3] | 92.9  [89.5-96.2] | 0.756  [0.661-0.851] | 19.2 [8.4-43.9] | 1.30E-12 | 43.2  [27.5-60.4] | 2.2  [0.8-5.4] |

hrHPV(+): HPV 16, 18, 31, 33, 35, 39, 45, 51, 52, 56, 58, 59, 66 and 68, positive for one or more of them. HPV16/18(+): positive results for HPV16 and (or) HPV18; non-16/18 hrHPV(+)：positive hrHPV results exclusive HPV16/18 positive; CISCER positive criteria: ΔCt*PAX1*≤6.6 and (or) ΔCt*JAM3*≤10.0; *PAX1*^m^ positive criteria: ΔCt*PAX1*≤6.6; *JAM3*^m^ positive criteria: ΔCt*JAM3*≤10.0; LBC_ASC-US_: the results of cytology were ASC-US and worse, including ASC-US, LSIL, ASC-H, HSIL and cervical cancer; LBC_LSIL_: the results of cytology were LSIL and worse, including LSIL, ASC-H, HSIL and cervical cancer; LBC_ASC-H_: the results of cytology were ASC-H and worse, including ASC-H, HSIL and cervical cancer; Abbreviations: CIN2+: cervical intraepithelial neoplasia (CIN) 2 or worse; CI: confidence interval; PPV: positive predictive value; NPV: negative predictive value; AUC: area under the curve; CISCER: *PAX1*^m^/*JAM3*^m^; *PAX1*^m^: the methylation of paired box gene (*PAX1*) gene; *JAM3*^m^: the methylation of junctional adhesion molecule 3 (*JAM3*) gene; LBC: liquid‑based thin‑layer cytology testing, (+), positive result; (−), negative result.
